# Early recognition of methicillin-resistant *Staphylococcus aureus* surgical site infections using risk and protective factors identified by a group of Italian surgeons through Delphi method

**DOI:** 10.1186/s13017-017-0136-3

**Published:** 2017-06-12

**Authors:** G. Sganga, C. Tascini, E. Sozio, S. Colizza

**Affiliations:** 1 Istituto Clinica Chirurgica, Divisione Chirurgia Generale e del Trapianto di Fegato, Università Cattolica del Sacro Cuore, Fondazione Policlinico Universitario A. Gemelli, Largo Gemelli, 8, 00168 Roma, Italia; 2 Prima Divisione Malattie Infettive, Azienda Ospedaliera dei Colli, Napoli, Italia; 3 U.O. Medicina d’Urgenza Universitaria, Azienda Ospedaliero-Universitaria Pisana, Pisa, Italia; 4 Master Sepsi in Chirurgia, Università Cattolica del Sacro Cuore, Roma, Italia

**Keywords:** Acute bacterial skin and skin structure infections, Surgical site infection, MRSA, Dalbavancin

## Abstract

**Background:**

Surgical site infections (SSIs) constitute a major clinical problem in terms of morbidity, mortality, duration of hospital stay, and overall costs. The bacterial pathogens implicated most frequently are *Streptococcus pyogenes* (*S. pyogenes*) *and Staphylococcus aureus* (*S. aureus*). The incidence of methicillin-resistant *S. aureus* (MRSA) SSIs is increasing significantly. Since these infections have a significant impact on hospital budgets and patients’ health, their diagnosis must be anticipated and therapy improved. The first step should be to evaluate risk factors for MRSA SSIs.

**Methods:**

Through a literature review, we identified possible major and minor risk factors for, and protective factors against MRSA SSIs. We then submitted statements on these factors to 228 Italian surgeons to determine, using the Delphi method, the degree of consensus regarding their importance. The consensus was rated as positive if >80% of the voters agreed with a statement and as negative if >80% of the voters disagreed. In other cases, no consensus was reached.

**Results:**

There was positive consensus that sepsis, >2 weeks of hospitalization, age >75 years, colonization by MRSA, and diabetes were major risk factors for MRSA SSIs. Other possible major risk factors, on which a consensus was not reached, e.g., prior antibiotic use, were considered minor risk factors. Other minor risk factors were identified. An adequate antibiotic prophylaxis, laparoscopic technique, and infection committee surveillance were considered protective factors against MRSA SSIs. All these factors might be used to build predictive criteria for identifying SSI due to MRSA.

**Conclusions:**

In order to help to recognize and thus promptly initiate an adequate antibiotic therapy for MRSA SSIs, we designed a gradation of risk and protective factors. Validation, ideally prospective, of this score is now required. In the case of a SSI, if the risk that the infection is caused by MRSA is high, empiric antibiotic therapy should be started after debriding the wound and collecting material for culture.

## Background

### Surgical site infections: background

The Food and Drug Administration (FDA) recently proposed a new classification of skin and soft tissue infections namely acute bacterial skin and skin structure infections (ABSSSIs) which comprise erysipelas, cellulitis, major subcutaneous abscesses, and wound infections, including surgical site infections (SSIs). An ABSSSI is a bacterial infection of the skin with a lesion area of at least 75 cm^2^, measured by redness, edema, or induration [1]. ABSSSIs constitute a significant burden for the healthcare system and their incidence is increasing. They have become a challenging clinical problem associated with high direct and indirect costs. Bacterial pathogens that commonly cause ABSSSIs include S. *pyogenes* and S. *aureus*, including a large number of methicillin-resistant *S. aureus* (MRSA) strains.

Given the associated morbidity, mortality, length of hospital stay, and overall direct and indirect costs, SSIs represent a significant problem [2–4]. Moreover, MRSA infections can have an even greater impact on hospital budgets and on patients’ health, since they are more severe, may lead to longer hospital stays, and are associated with higher mortality.

Despite progresses in prevention, SSIs remain one of the most common adverse events in hospitals, accounting for 11 to 26% of all healthcare-associated infections [5]. In Italy, the rate of SSIs ranged from 5.4 to 12.8%, although recent studies have shown a reduction to 5%, [6, 7]. Surgical patients can develop several types of post-operative infection, with wound infections being the most common. The specific surgical procedures for each specialty are associated to different percentages of SSIs [8]. These complications add 10–20% additional extra costs to the total hospital bill [9].

In the USA, for any given type of operation, the development of a wound infection will approximately double the cost of hospitalization. These infections lead to 80,000 deaths per year and are associated with an annual treatment cost of US$2 billion [10]. A similar scenario has been found in Italy, where nosocomial infections occur in 500,000 out of 8,000,000 hospital admissions per year [11].

SSIs caused by S. *aureus* may be life threatening (estimated mortality rate 5%) and account for more than two extra weeks of hospitalization and around US$50,000 extra cost [12]. Rates of MRSA infection are increasing dramatically, and there is an urgent need to reduce hospitalizations and costs and implement more effective outpatient management and treatment strategies. Since cost containment and cost-efficient patient management are top priorities today, for the whole healthcare system, many hospitalized patients with SSIs could be safely treated, as outpatients, with current options of intravenous antibiotic therapy. This would reduce costs for hospitalization and could also improve patients’ outcome and satisfaction.

### Diagnosis-related groups and the incidence of surgical site infections

The exact incidence of SSIs is difficult to determine. In fact, the Diagnosis-Related Group (DRG) system underestimates the rate of SSIs, due to a very early discharge of surgical patients. Hospitals without a surveillance system report infection rates of less than 5%. This figure probably underestimates the problem, mostly because many SSIs are diagnosed after the patient has been discharged from hospital [8]. For example, Petrosillo et al. described that SSIs occurred in 5.2% out of 4665 patients and that, while 148 SSIs (61.4%) were diagnosed during the patients’ stay in hospital, 93 (38.6%) were recorded within 30 days after discharge [13]. This is comparable to the 34.8% result found in another Italian study on general surgery patients reported by Fiorio et al. [14]. However, in another similar surveillance study, higher post-discharge rates of SSI were found [15]. Marchi et al. reported data on non-prosthetic surgery from the Italian SSI surveillance program for the period 2009 to 2011, based on 60,460 operations from 355 surgical wards: SSIs were observed in 1628 cases (2.6%), with 60% of these being diagnosed on day 30 post-discharge surveillance [16].

Thus, up to 60% of SSIs are diagnosed after discharge from hospital, and this trend is increasing as the length of post-operative hospital stay is getting shorter and the number of 1-day surgery procedures is increasing [17].

According to current literature, active infection surveillance is useful in reducing the incidence of SSIs through surveillance-induced infection control efforts [18]. Operations performed in hospitals with at least 2 years of surveillance showed a 29% lower risk of SSIs, confirming that surveillance is a protective factor in preventing SSIs [16].

### Classification of surgical site infections

The identification of SSIs involves interpretation of clinical and laboratory findings. The Centers for Disease Control and Prevention (CDC) National Nosocomial Infections Surveillance (NNIS) system has developed standardized surveillance criteria for defining SSIs [19]. Using these criteria, SSIs are classified as either incisional or organ/space. Incisional SSIs are further divided into those involving only skin and subcutaneous tissue (superficial incisional SSIs) and those involving deeper soft tissues of the incision (deep incisional SSIs). Organ/space infections are not included in ABSSSIs.

### Predominant pathogens in surgical site infections

According to the NNIS system reports, the most commonly encountered pathogens in SSIs are Gram-positive cocci, particularly *Staphylococcus aureus* (*S. aureus*), coagulase-negative staphylococci (CoNS), *Streptococcus pyogenes* (*S. pyogenes*), and *Enterococcus* spp., followed by *Escherichia coli*, *Pseudomonas aeruginosa* and *Enterobacter* spp. [20]. The source of pathogens is often the endogenous flora of the patient’s skin, mucous membranes, or hollow viscera [21]. Microorganisms of endogenous flora are usually aerobic Gram-positive cocci (e.g., staphylococci), but may also include fecal flora (e.g., anaerobic bacteria and Gram-negative aerobes) [22].

Exogenous sources of pathogens involved in SSIs include surgical personnel (especially members of the surgical team), the operating room environment (including air), and all tools, instruments, and materials brought to the sterile field. Exogenous flora consists primarily of aerobes, especially Gram-positive organisms (e.g., staphylococci and streptococci) [23].

Besides *S. aureus* and CoNS, *Staphylococcus epidermidis* may be responsible for severe infections following implantation of any prosthesis in general surgery, cardiac surgery, orthopedics, etc. Such infections may require removal of the prosthesis.

Table 1 lists the more frequent pathogens, according to surgical procedure.

**Table 1:** Most frequently encountered pathogens, according to the surgical procedure [8]

Type of surgery	Likely pathogens
Placement of grafts, prostheses, or implants	*S. aureus*, coagulase-negative staphylococci
Cardiac	*S. aureus*, coagulase-negative staphylococci
Neurosurgery	*S. aureus*, coagulase-negative staphylococci
Breast	*S. aureus*, coagulase-negative staphylococci
Ophthalmic (limited data; anterior segment resection, vitrectomy, and scleral buckling)	*S. aureus*, coagulase-negative staphylococci, streptococci, Gram-negative bacilli
Orthopedic (total joint replacement, closed fractures/use of nails, plates, other internal fixation device, functional repair without implant/device trauma)	*S. aureus*, coagulase-negative staphylococci, Gram-negative bacilli
Non-cardiac thoracic (lobectomy, pneumonectomy, wedge resection, other non-cardiac mediastinal procedures), closed tube thoracotomy	*S. aureus*, coagulase-negative staphylococci, *S. pneumoniae*, Gram-negative bacilli
Vascular	*S. aureus*, coagulase-negative staphylococci
Appendectomy	Gram-negative bacilli, anaerobes
Biliary tract	Gram-negative bacilli, anaerobes
Colorectal	Gram-negative bacilli, anaerobes
Gastroduodenal	Gram-negative bacilli, streptococci, oropharyngeal anaerobes (e.g., peptostreptococci)
Head and neck (mainly procedures with incision through oropharyngeal mucosa)	*S. aureus*, streptococci, oropharyngeal anaerobes (e.g., peptostreptococci)
Obstetric and gynecological	Gram-negative bacilli, enterococci, group B streptococci, anaerobes
Urological	Gram-negative bacilli

*S. aureus *Staphylococcus aureus

### Staphylococcus aureus in surgical site infections

*S. aureus* is consistently the leading cause of nosocomial infections, including SSIs. The incidence of MRSA strains is rising dramatically, and the amount of hospitalizations has more than doubled, in the past decade. Remarkably, MRSA is the main pathogen causing SSIs in many academic and community hospitals [24]. This represents a significant, independent risk factor for adverse clinical and economic outcomes. Kirkland et al. estimated that the excess hospital costs associated with MRSA SSIs ranged from US$3089 to US$35,367 [3]. Engemann et al. found that, among surgical patients, those with MRSA infections were hospitalized 5 days longer than those with methicillin-sensitive *S. aureus* (MSSA) infections [25]. Hospital charges for MRSA patients were also US$62,908 greater than for patients without an infection and US$40,000 greater than for patients with MSSA infections.

Other studies support the findings that patients with MRSA infections require more health-care resources than patients with MSSA infections [26]. Weigelt et al. determined that the risk-adjusted attributable increase in duration of hospitalization was approximately 1 day and, in terms of costs for the hospitalization, over US$1000. They also found that significant independent risk factors, increasing costs, and time spent in hospital for SSIs due to MRSA, included illness severity, transfer from another healthcare facility, a previous hospital admission (30 days), and other polymicrobial infections (*p* < 0.05) [27].

### Methicillin-resistant *Staphylococcus aureus*

MRSA is a versatile and dangerous bacterial pathogen that shows virulence, antibiotic resistance, and survival fitness [28]. Selection pressure, exercised by broad-spectrum antibiotic treatment and cross-transmission through healthcare workers’ hands, facilitates its spreading. Hence, profiles to identify patients at high risk of MRSA carriage might improve prevention of MRSA infections because MRSA carriers, without symptomatic infection, are an important pathogen reservoir [29].

Efforts should be made to identify patients at risk of colonization and subsequent infection by multi-drug-resistant microorganisms such as MRSA, and to control hospital-acquired SSIs. Remarkably, surveillance systems, even without any specific intervention, have been associated with a reduction in SSIs [30]. Because all authors do not recommend a universal rapid MRSA screening, other methods to pick out patients at high risk of MRSA colonization and subsequent SSIs could be very useful.

Harbart et al. found that age 75 years or older, previous hospitalization (in the preceding 12 months), and recent antibiotic therapy (in the preceding 6 months) were the strongest risk factors for MRSA colonization in surgical patients, then confirmed in a validation cohort [31].

Applying the Delphi method, we recently identified the most common risk factors for colonization and/or infection by MRSA as being patient from a long-term care facility, previous hospitalization (within the preceding 30 days), Charlson score >5, chronic obstructive pulmonary disease and thoracic surgery, antibiotic therapy with beta-lactams (especially cephalosporin and carbapenems) and/or quinolones in the preceding 30 days, age 75 years or older, current duration of hospital admission >16 days, and prosthetic implant surgery. Furthermore, protective factors were an adequate antibiotic prophylaxis, laparoscopic surgery, and the presence in the hospital of an active surveillance program for infection control [8].

### Therapy for methicillin-resistant *Staphylococcus aureus*

Clinical MRSA isolates show a decreased susceptibility or increased resistance to anti-MRSA drugs. Thus, treatment of ABSSSIs is now challenged by toxicity, few oral options, and greater need for hospitalization and its associated costs [32].

The Infectious Diseases Society of America (IDSA) recommended beta-lactam or clindamycin for mild/moderate non-purulent ABSSSIs, and vancomycin plus piperacillin/tazobactam for severe non-purulent ABSSSIs treatments [33]. In purulent ABSSSIs, doxycycline or trimethoprim/sulfamethoxazole should cover MRSA for moderate cases and vancomycin, daptomycin, linezolid, or ceftaroline in severe cases. Antimicrobials available in Italy for treating ABSSSIs with activity against MRSA and other resistant Gram-positive pathogens include vancomycin, teicoplanin, daptomycin, linezolid, and ceftaroline. Of these antimicrobials, only linezolid is available in an oral formulation.

Table 2 reports the main characteristics of anti-MRSA drugs for SSIs. Some of these drugs have significant potential toxicity (e.g., myopathy from daptomycin, renal function impairment from vancomycin, bone marrow suppression from linezolid) and drug interactions (e.g., linezolid and selective serotonin re-uptake inhibitors). While doxycycline and trimethoprim/sulfamethoxazole are active against MRSA, their activity against beta-hemolytic streptococci is poor, limiting their use as monotherapy for ABSSSIs.

**Table 2:** Main characteristics of the antibiotics used for the treatment of SSIs due to MRSA [8]

Antibiotic	Bactericidal activity; pharmacodynamics; anti-biofilm activity	Route of administration	Doses	Adverse events	Interactions	Cost (for a 70 kg person)
Teicoplanin	Bactericidal with low MIC; time-dependent; None	iv, im	7-10 mg/Kg once daily, loading dose	Renal toxicity	None	€50-70/day
Vancomycin	Bactericidal with low MIC; time-dependent; None	iv	1 g twice daily, 500 mg four times a day	Renal toxicity	Other nephrotoxic drugs	€5/day
Daptomycin	Bactericidal; concentration-dependent; Yes	iv	4-6 mg/kg	Myotoxicity	Statins	€80-120/day
Linezolid	Bacteriostatic; time-dependent; None	iv, oral	1200 mg once daily	Bone marrow toxicity, neuropathy, serotoninergic syndrome	SSRIs	€120/day
Tigecycline	Bacteriostatic; time-dependent; Partial	iv	50 mg twice daily; 100 mg loading dose	Nausea, vomiting, pancreatitis	None	€120/day
Ceftaroline	Bactericidal; time-dependent; None	iv	600 mg twice daily	Rash	None	€96/day
Dalbavancin	Bactericidal; concentration-dependent; Yes	iv	1000 mg day 1, 500 mg after 7 days; or 1500 mg one-shot	No	None	NA
Cotrimoxazole	Bactericidal; time-dependent; None	iv, oral	800/160 mg 3 times a day	Anemia	None	€15/day
Rifampin	Bactericidal; time-dependent; Yes	iv, oral	600 mg once a day	Liver toxicity	Several	€6/day

*iv* intravenous, *im* intramuscular, *SSRIs* selective serotonin re-uptake inhibitors

Dalbavancin, a novel lipoglycopeptide antibiotic active against both MSSA and MRSA, was approved by the FDA in May 2014 and by the European Medicines Agency (EMA) in February 2015 for the treatment of ABSSSIs caused by susceptible Gram-positive organisms [34]. Several clinical trials have demonstrated its tolerability, efficacy, and non-inferiority compared to standard therapy for ABSSSIs. The DISCOVER 1 and DISCOVER 2 studies showed that once-weekly intravenous dalbavancin was not inferior to twice-daily intravenous vancomycin, followed by oral linezolid for the treatment of ABSSSIs. Adverse events were reported less frequently in patients treated with dalbavancin, than in those treated with vancomycin-linezolid [35]. Dalbavancin’s high-protein binding and prolonged half-life allow for easily and consistently attainable therapeutic levels. It has a well-established activity against the Gram-positive organisms commonly involved in superficial and deep SSIs, including MRSA and other multidrug-resistant pathogens, and the MIC_90_ values for these organisms have remained stable over the past decade.

Dalbavancin, as an anti-MRSA agent, displays advantageous factors, such as bactericidal effect, full activity on biofilm [36], which could be increased by combination with rifampicin, lack of drug-drug interactions, lack of renal toxicity, and no adverse events associated with an early discharge from hospital, because of its prolonged half-life. Indeed, the most unique feature of dalbavancin is its once-weekly dosing, previously approved as a 1000 mg dose followed by 500 mg 1 week later, and then the one-shot, single-dose of 1500 mg, recently approved by the EMA [37].

The aim of this study was to create a score for the early recognition of SSIs caused by MRSA in order to facilitate the prompt initiation of adequate antibiotic therapy.

## Methods

After a systematic literature review on SSIs (randomized clinical trials, case-control studies, recommendations and case reports), we proposed some major and minor risk factors, and protective factors, drawn from the literature and identified in a previous work [8], to an expert board of general and specialist surgeons.

### Delphi methodology

The Delphi method is a widely used technique to define standards, therapies, and care procedures based on the opinions of groups of experts [38–40].

An algorithm method may be useful for identifying patients without risk factors (high negative predictive model) but may be less efficient at identifying the true positives (low positive predictive value). With the Delphi method, each participant expresses his or her opinion anonymously, contributing to create the expert opinion of the entire group. The participants are asked to respond to a questionnaire, divided into statements, and independently assign a score ranging from 1 (which corresponds to “maximum disagreement”) up to 5 (which corresponds to “absolute agreement”).

We considered a positive consensus when more than 80% of the participants agree with the statement proposed and negative consensus when >80% of the participants disagree with it. In other cases, a consensus was not reached.

Here, 228 surgeons, members of the Italian Society of Surgeons, were asked to find a possible consensus on risk factors for MRSA, using the Delphi method [38–40]. The questions, based on a review of the literature and our previous research, concerned major and minor risk factors for and protective factors against SSIs due to MRSA.

## Results

Having identified possible major and minor risk factors for MRSA SSIs, as well as potential protective factors, we determined the level of consensus regarding these factors among a large group of Italian surgeons using the Delphi method. Consensus was achieved when more than 80% of the group agreed (positive consensus) or disagreed (negative consensus) on a statement proposed.

Table 3 reports the submitted risk and protective factors with the rate of agreement. The questions were rated as major and minor risk factors for SSIs due to MRSA.

**Table 3:** Risk factors for and protective factors against MRSA SSIs with the level of agreement obtained by the Delphi method

Risk factors for SSIs due to MRSA	Percentage of consensus
Major risk factors for MRSA SSIs	
Signs and severity of sepsis	Consensus (>80%)
Colonization by MRSA	Consensus (>80%)
Age > 75 years	Consensus (>80%)
Duration of hospitalization > 2 weeks	Consensus (>80%)
Previous treatment with antibiotics, from 30 days to 12 months	No consensus (<80%)
ICU admission in the previous 12 months	No consensus (<80%)
Any prosthetic surgery	No consensus (<80%)
Previous admission to hospital (6 months) and/or rehabilitation structure	No consensus (<80%)
Minor risk factors for MRSA SSIs	
Diabetes (HbA1c > 7%)	Consensus (>80%)
Obesity (BMI > 30)	No consensus (<80%)
Steroids and immunosuppressive treatment	No consensus (<80%)
Previous hospital admission from 30 days to 6 months	No consensus (<80%)
Renal insufficiency	No consensus (<80%)
Chronic obstructive pulmonary disease	No consensus (<80%)
Other antibiotic therapy from 30 days to 6 months	No consensus (<80%)
Surgical operation lasting more than 3 h	No consensus (<80%)
Protective factors for MRSA SSIs	
Adequate antibiotic prophylaxis	Consensus (>80%)
Laparoscopic technique	Consensus (>80%)
Hospital with an Infection Surveillance Committee	Consensus (>80%)

*ICU* intensive care unit, *HbA1c*, glycated hemoglobin, *BMI* body mass index

A positive consensus was reached on the fact that sepsis, >2 weeks spent in hospital, age >75 years, and colonization by MRSA were major risk factors for MRSA SSIs (Table 4). The board also agreed that diabetes was a minor risk factor for MRSA SSIs. Other possible risk factors, on which a consensus was not reached, i.e., antibiotic use in the preceding year, ICU admission in the preceding year, prosthetic surgery, admission to hospital or rehabilitation facility within the preceding 6 months, obesity, steroids and immunosuppressive treatment, renal insufficiency, chronic obstructive pulmonary disease, and surgery lasting more than 3 h, were considered minor risk factors. An adequate antibiotic prophylaxis, laparoscopic technique, and infection committee surveillance were considered strong factors protecting against MRSA SSIs (Table 4).

**Table 4:** Suggested score to assess the risk of MRSA SSIs

MRSA SSIs risk and protective factors	
Sepsis signs and severity	Major risk factor
Duration of hospitalization > 2 weeks	Major risk factor
Age > 75 years	Major risk factor
Colonization by MRSA	Major risk factor
Diabetes (HbA1c > 7)	Major risk factor
Procalcitonin dosage >3 mg/dl	Minor risk factor
Previous admission in hospital (6 months) and/or rehabilitation structure	Minor risk factor
Previous treatment with antibiotics, from 30 days to 12 months	Minor risk factor
ICU admission during the previous 12 months	Minor risk factor
Any prosthetic surgery	Minor risk factor
Obesity (BMI > 30)	Minor risk factor
Steroids and immunosuppressive treatment	Minor risk factor
Previous hospital admission from 30 days to 6 months	Minor risk factor
Renal insufficiency	Minor risk factor
Chronic obstructive pulmonary disease	Minor risk factor
Other antibiotic therapy from 30 days to 6 months	Minor risk factor
Surgical procedure lasting more than 3 h	Minor risk factor
Adequate antibiotic prophylaxis	Major protective factor
Laparoscopic technique	Major protective factor
Hospital with an Infection Surveillance Committee	Major protective factor

*ICU* intensive care unit, *HbA1c* glycated hemoglobin, *BMI* body mass index

These factors should be validated in prospective studies, for better identification of patients at higher risk of developing MRSA SSIs or earlier identification of patients who already have an SSI, due to MRSA. To promptly start an appropriate, effective antibiotic therapy promptly, we proposed to identify major and minor risk and protective factors, to assess the risk of MRSA SSIs, as shown in Table 4, we merged results from a review of the literature and the Delphi consensus results described here. We think that these factors should be evaluated for predicting the need to start antibiotic therapy, given the high risk of the patient having a MRSA SSI.

## Discussion

ABSSSIs have become a challenging clinical issue, associated with high direct and indirect costs. SSIs are an important subgroup of ABSSSIs and are associated with high rates of morbidity. Among other pathogens, MRSA is prevalent in ABSSSIs and in SSIs. Indeed, the rates of MRSA infection and related hospitalizations are increasing dramatically and MRSA has emerged as the most common cause of purulent infections. Besides, MRSA infection is a risk factor for subsequent hospitalization and death. In the management of surgical patients, it could therefore be very important to recognize if there are risk factors for the development of SSI caused by MRSA.

Through a systematic review of the literature and the consensus of 228 Italian surgeons, obtained by the Delphi method, we made an effort to identify and quantify the importance of risk factors for and protective factors against MRSA SSIs. Our primary aim was to facilitate the early recognition of SSIs caused by MRSA, which leads to promptly start an adequate antibiotic therapy (Table 4). These risk and protective factors can be rapidly validated in retrospective and/or perspective studies, in order to have an instrument, for surgeons and physicians, to identify patients with a suspected MRSA SSI. However, our propositions are not an alternative to current, recommended measures of hygiene and appropriate antibiotic prophylaxis.

In the case of a SSI, after debridement of the wound and taking samples of the material obtained from the wound for culture, empiric antibiotic therapy should be started, especially if the risk of a MRSA infection is high. There is, however, an urgent need to reduce hospitalization, through the use of more effective outpatient treatment strategies that can reduce costs and improve patients’ outcomes and satisfaction. Because early discharge is almost always recommended for surgical patients, dalbavancin might be a beneficial addition to the therapeutic armamentarium for the treatment of SSIs, since its use does not prolong the time of the hospital stay. In fact, dalbavancin, with its long half-life, can be administered also in the case of programmed discharge of a patient.

## Conclusions

SSIs due to MRSA are associated with considerable morbidity and mortality, as well as being a heavy financial burden on the healthcare system. Through a systematic review of the literature and the consensus of 228 Italian surgeons, obtained by the Delphi method, we suggest major and minor risk and protective factors to facilitate the early recognition of SSIs caused by MRSA in order to promptly start an appropriate antibiotic therapy.

## Abbreviations

ABSSSIs: Acute bacterial skin and skin structure infections
Im: Intra-muscular
Iv: Intra-venous
MRSA: Methicillin-resistant *Staphylococcus aureus*
MSSA: Methicillin-sensitive *Staphylococcus aureus*
SSI: Surgical site infection
